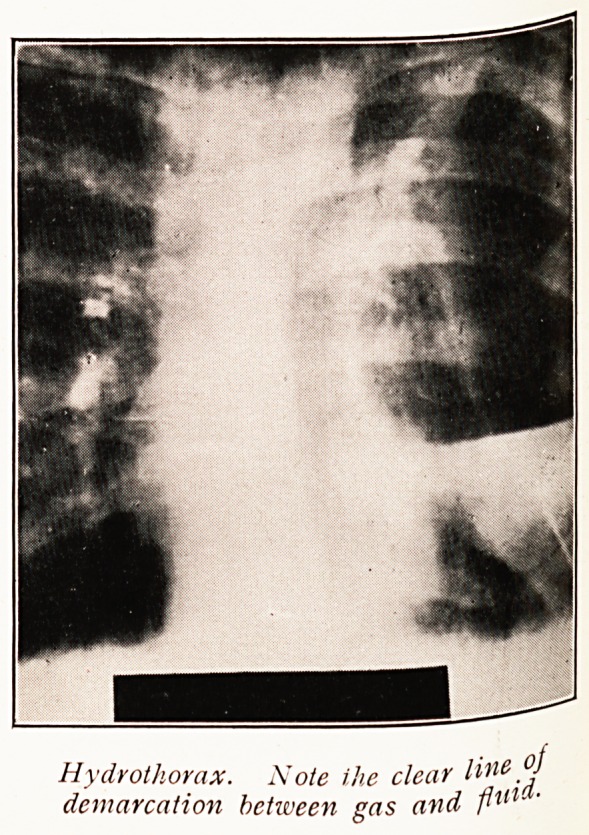# Some Problems in Pulmonary Disease, with Special Reference to Radiography

**Published:** 1925

**Authors:** H. H. Carleton

**Affiliations:** Assistant Physician to the Bristol General Hospital


### SOME PROBLEMS IN PULMONARY DISEASE, WITH SPECIAL REFERENCE TO RADIOGRAPHY.

BY

H. H. CARLETON, M.A., M.D. (Oxon.), M.R.C.P.,

*Assistant Physician to the Bristol General Hospital.*

---

THE extent of information which may be derived from pulmonary radiography, also the limitations of this method, are worthy of consideration. Assuming that the technique for the production of satisfactory radiographs leaves nothing to be desired, difficulties of correct interpretation are responsible for a certain sense of disappointment, and tend to diminish the usefulness of pulmonary radiographs for many clinicians. Over-interpretation, *i.e.* the drawing of deductions which are logically unsound, from radiographs alone, is responsible for some of this disappointment and creates sceptics.

It should hardly be necessary to emphasise that radiography of lungs is a method to be used in conjunction with clinical signs and symptoms ; by itself the method is incomplete, and should seldom form the sole basis of an opinion. For instance, it is obvious that a single skiagraph showing well-marked shadows of pulmonary tuberculosis, can give no information as to the activity of the disease. On the other hand, several skiagraphs taken at intervals may show, by virtue of alteration in the shadows, that active changes are in progress.

One point on the technical side of pulmonary radiography : it is impossible to get good pictures showing satisfactory detail in lungs except with practically instantaneous exposures. It is not enough that a patient can hold his breath for a few seconds. Heart movements cannot be inhibited similarly ; they are considerable, and are sufficient to blur the picture. Personally, I have never seen a good lung picture in which the exposure exceeded about half a second. In taking a chest for lung disease we do not want to see the structure of the ribs. Over-exposed, flat and blurred films are responsible for much of the dissatisfaction expressed by some as to the value of pulmonary radiography. A second point on the technical side : one should always insist that the picture shall include the whole of the lungs. Small films which do not show the whole of the intercostal spaces are valueless. It is in the peripheral parts of the intercostal spaces that we look for the mottling characteristic of pulmonary tuberculosis. Thirdly : it is desirable to take all skiagraphs in a standard position. If the position of the tube is varied, considerable alteration of appearances at the apices results and, further, it becomes impossible to compare satisfactorily skiagraphs of the same patient taken at intervals.

It needs to be emphasised that there is no such thing as a normal chest ; in other words, appearances differ widely in perfectly healthy people. The difference is chiefly in the amount of fibrous tissue. A good radiograph, by reason of the detail, will show proportionately more conspicuous shadows of the bronchial tree than will a flat over-exposed negative. If this is not recognised we shall be confronted with the suggestion that every patient, who obtains a good skiagraph, is suffering from fibrosis, which is manifestly absurd.

Pulmonary fibrosis is one of the problems I wish to raise for discussion.

I may say at the outset I am a complete disbeliever in the interpretation of striation in pulmonary radiographs as indicative of pathological fibrosis. Such a conclusion from radiographic shadows is an unjustifiable deduction.

The clinical signs of fibrosis are chest deformity, in the form of shrinkage or falling in of the chest wall, cough and dyspnœa. Shrinkage includes, of course, displacement of internal viscera.

To appreciate all that is involved one must turn to the essential significance of cough as a symptom. Why does anyone cough? Fundamentally it would appear the cough is a physiological response to some degree of obstruction in the air passages anywhere between and including air vesicles and larynx. We may for the moment neglect certain reflex causes of cough, *e.g.* wax in the ears.

I believe that in every case of pulmonary tuberculosis cough is a constant symptom, though it may be insignificant and denied by the patient unless the matter is carefully inquired into.

The invasion of the wall of a bronchiole by a spreading tubercle results in an obstruction to the air-way from a neighbouring air vesicle, with resulting broncho-pneumonic catarrh and the formation of a yellow tubercle. This is productive of a short spasmodic cough. The X-ray appearance is stippling in the lung.

But this stippling is not pathognomonic of tubercle, for it occurs just as obviously in pneumokoniosis and even in simple bronchopneumonia. The actual truth seems to be that stippling is an index of terminal vesicular catarrh, —bronchopneumonia, if you prefer the term—and this is the only deduction that may be drawn from X-ray appearances.

In cases of pneumokoniosis the process is extremely chronic, and the X-ray appearances are characteristic, in that wide-spreading lesions, originally bronchopneumonic areas, come to be pervaded by dense fibrous plaques which are very obvious in skiagraphs. Such huge masses are seldom seen in pulmonary tubercle; because an extensive invasion with the tubercle bacillus usually brings death long before the protective response in the form of fibrosis can appear to the high degree seen in pneumokoniosis. One is forced, then, to the conclusion that pulmonary tuberculosis and pneumokoniosis yield shadows that cannot with certainty be distinguished by X-ray appearances alone.

Radial peribronchial striation causes no deformity of chest wall, no displacement of viscera, no obstruction to the air-way, and therefore, *per se*, no cough. It is usually not a sign of pulmonary fibrosis in any pathological sense, but merely an index of increasing years, and comparable to the gradual development of arteriosclerosis.

One would not be so keen to raise this question were it not that the fallacy is costing the country thousands of pounds a year in pensions to ex-soldiers, in addition to which there is the serious mental effect on men who have been taught to consider themselves invalids on no better evidence than the presence of striæ in a radiographic picture.

The position of the tracheal shadow is always worthy of attention; it should be visible in every skiagraph, and normally, of course, occupies the mid-line. Its upper part is occasionally displaced by a goitre to such an extent that the details of the larynx are visible. Deviation of the lower part of the tracheal shadow is often a sign of pulmonary fibrosis. Naturally deviation indicates asymmetrical fibrosis. It is a frequent accompaniment of pulmonary tuberculosis or pneumokoniosis, and is an index of chronicity. Sometimes a patient gives only a short history of failure of health, suggesting that the present illness is the first breakdown from tuberculosis. However, well-marked deviation of the trachea indicates that probably he has been the subject of a previous attack, possibly years before. This deduction will carry some weight also in prognosis, because it indicates that the patient possessed a relatively good resistance in the past, and may reasonably be expected again to respond well to treatment.

Incidentally the degree of tracheal displacement is a useful check on manometric readings, when judging the mechanical effects of increasing intrathoracic pressure in treatment by artificial pneumothorax. The trachea shows the effects of pressure more readily than the mediastinum generally, at any rate the displacement of the trachea can be more readily detected. It is possible to maintain good pulmonary collapse without displacing the trachea. If this rule is observed some of the difficulties and discomforts associated with the maintenance of an artificial pneumothorax, particularly on the left side, can be avoided.

Gross displacements of the mediastinum are usually obvious. Unilateral, uniform opacity, associated with mediastinal displacement is generally diagnostic of fluid. Sometimes malignant disease, *e.g.* lymphosarcoma, may produce a uniform opacity on one side of the chest, but such a condition does not usually displace the mediastinum.

The X-ray appearances of fluid in the chest deserve further mention. A simple effusion never presents a clearly-defined upper level. The appearance of fluid exhibiting a clear-cut, straight line is pathognomonic of gas plus fluid in the pleural cavity. The free mobility of the fluid when gas is present is very characteristic in a screen examination. Hydrothorax occurs rather frequently in cases of artificial pneumothorax and should be looked for. Sometimes such fluid undergoes spontaneous absorption.

I know of no X-ray appearances by which we may distinguish between serous effusion and pus. The distinction is to be based on other evidence.

Perhaps the grossest displacements of the heart and mediastinum are associated with chronic fibroid phthisis of long duration. Such displacements are always associated with well-marked falling in of the intercostal spaces on the affected side. The heart may be drawn over to such an extent that there is no heart shadow lying in front of the vertebral column.

I wish now to pass to a discussion of appearances in the region of the hilum of the lung, together with the shadows of scattered intrapulmonary glands. Hilum shadows are present in early childhood and increase with age, as a consequence of the relative increase in fibrous tissue. Even dense hilum shadows are without pathological significance *per se*, and should never form the basis of a diagnosis of fibrosis. This has already been discussed in dealing with fibrosis; but the presence of clearly-defined glands at the hilum and in the substance of the lung requires more consideration. Personally I regard the presence of opaque glands very much in the same light as I view the von Pirquet reaction. They represent in many cases old obsolete reactions to past infections. Everyone acquires infection with the tubercle bacillus and undergoes immunising reactions, in which lymphatic glands play a prominent part. The presence of opaque glands must not be interpreted as evidence of broken-down resistance past or present. Supposing the glandular barrier fails, infection is carried by the lymphatics, interlobular, peribronchial or perivascular, to the peripheral parts of the lung distal to the intrapulmonary glands. Here bronchopneumonic lesions develop, and are seen as characteristic stippling in the peripheral parts of the intercostal spaces. We are led to the following position if my interpretation be correct, that opaque glands *per se* are not to be taken as evidence of clinical tuberculosis. But if peripheral stippling is also present the glandular

PLATE XIII.

*Normal lung with unusually well-marked striation.*

*Pulmonary fibrosis from a case of silicosis. Note falling together of intercostal spaces.*

*Silicosis showing plaques of fibrous tissue.*

*Pneumothorax.*

### PLATE XIV.

*Large effusion. Note displacement of mediastinum.*

*Malignant disease. Note mediastinum is not displaced, owing to adhesions*

*Acute tuberculosis.*

*Hydrothorax. Note the clear line of demarcation between gas and fluid.*

barrier has broken down. It should be emphasised that peripheral stippling is the earliest radiographic sign of pulmonary tuberculosis. It is best seen in cases of miliary tuberculosis.

Turning now to the appearances of cavities in the lung in skiagraphs, it is by no means uncommon to see reports stating that cavities are present in cases which exhibit no clinical signs of cavitation. The truth seems to be that while all cavities are visible in good radiographs, there are a number of shadows which by their enclosed outline or roughly circular conformation may suggest cavities. The origin of these misleading shadows is quite uncertain. I think that some of them, at any rate, are formed by the churning action of mixed cardiac and respiratory movements upon plastic lymph thrown out on the surface of the lung. It will be a safe rule never to diagnose the presence of a cavity on X-ray appearances alone. Of course, thick-walled cavities may leave no doubt as to their nature, but in such cases other clinical signs will correspond.

The accurate localisation of cavities by X-rays is important in relation to certain forms of treatment which will be referred to later.

One important point in diagnosis emerges from the consideration of radiographs, namely that pulmonary tuberculosis even in its early stages is by no means always, I might almost say usually, the apical disease that we are taught to believe. Discussions, therefore, about alleged mechanism of apical infections do not really matter much. Perhaps it is true to say that the detection of the disease by physical examination is easier at the apices. Further, in the comparatively cramped space of the apices the pathological process early in the disease produces more crowded lesions and therefore relatively more destruction than in the more spacious areas of lung remote from the apex.

Pulmonary conditions other than those mentioned produce characteristic appearances. For instance, emphysema produces thin pictures with wide spacing of the ribs, the latter taking up a horizontal position.

Bronchiectasis is not, as a rule, well seen by X-rays unless there is a preliminary instillation of some radio-opaque substance, *e.g.* lipiodol. I have no experience as to the effects of aspirating nebulised opaque fluids for this purpose. The practical point with reference to the radiography of bronchiectasis is that unilateral cases can and should, I think, be treated by artificial pneumothorax. The benefit to the patient by this method in unilateral cases may be very real. Sputum may be reduced from a matter of, say, 6 oz. to an entirely negligible quantity. At the same time the treatment of bronchiectasis by this method is not free from risk. Only recently I have had a case in which, after an apparently quite successful induction of pneumothorax an empyema occurred, evidently from the breaking down of adhesions. As far as my own personal experience goes I have had no similar instance of infection following induction of artificial pneumothorax in tuberculous cases.

Turning more particularly to the value of X-rays as an adjunct to treatment, the problem of artificial pneumothorax calls for consideration. The indications for treatment by this method are a high degree of activity in unilateral cases. The value of X-rays in the selection of cases is quite obvious. However, cases in which the possibility of treatment by artificial pneumothorax arises are seldom truly unilateral. The problem presents itself in the following light: Is the proportion of active disease so overwhelming in one lung that, if the said lung can be immobilised, a very high proportion of existing toxæmia can be cut out, thus enabling the system to cope with any small residual amount of disease which may be present in the relatively sound lung?

In the selection of cases radiographs will indicate those which are quite unsuitable, either by reason of the bi-lateral extent of disease or by showing up the signs which go to indicate extensive adhesion. The latter may be presumed to exist where gross displacements of the mediastinal structures and the falling in of the intercostal spaces are present. Further, prior to induction of artificial pneumothorax it is desirable to know whether one is likely to meet with an area of normal pleura free from adhesion. Here, again, the value of X-rays is perfectly clear.

While for purposes of continuous treatment by artificial pneumothorax cases must be selected on the unilateral basis, the same desideratum does not apply to the use of the method as a hæmostatic. Artificial pneumothorax is one of the most efficient means of controlling hæmoptysis. The one point of prime importance is that we shall know from which lung the hemorrhage is occurring. It matters little or nothing that the opposite lung be free from disease, because in the ordinary way the treatment will not be persevered in once the danger of further bleeding is past. The advantage of artificial pneumothorax as a mode of arresting hemorrhage is that in the first place it prevents the spread of blood by means of the bronchial tree to parts of the lung hitherto not infected. The second great advantage is that nursing is greatly facilitated. The patient can be turned and moved about with safety, and food can be administered with much greater freedom than can be done in the case of treatment of hæmoptysis by older methods.

The appearance of pulmonary cavities in radiographs has already been discussed. From the surgical standpoint the localisation of cavities by X-rays is of considerable importance. The successful drainage of pulmonary abscesses is a case in point.

On the Continent cases with well-marked cavities are not infrequently treated by local compression where general compression of the lung by artificial pneumothorax cannot be achieved owing to adhesion. The success of such local surgical procedures is largely governed by radiographic records.

### THE PHTHINOID CHEST.

All of us are familiar with individuals presenting that poor type of physique usually designated the phthinoid chest: flat in front, kyphotic behind, narrow transversely, and long from above downwards, possessing poor expansion and cubic capacity. This type of chest has been associated with consumption in the minds of clinicians from the time of Aretaeus.

The physical signs on examination and the general appearance of the patient are so suggestive that they frequently lead, to say the least of it, to a premature diagnosis of pulmonary tuberculosis.

In my opinion the genesis of the phthinoid chest is to be found in enteroptosis. The subjects of this type of deformity practically invariably suffer from enteroptosis and a narrow " dropped heart " well displayed in radiographs.

I suggest that it is the general dropping of the abdominal contents which pulls down the lungs and diaphragm in their wake. In consequence expansion in the upper part of the chest can only occur by carrying and overcoming the overload of the abdominal contents. This is more than the enfeebled frame is equal to. The lungs at the apices, therefore, become permanently deflated and the breath sounds take on a bronchial character. The latter, associated with poor resonance and poor expansion, leads to the frequent diagnosis of " infiltration at both apices," a deduction which is quite unsound.

I should like to state in emphatic terms that in the case of a patient with a phthinoid chest no diagnosis of pulmonary tuberculosis should be made in the absence of post-tussic crepitations or of unquestionable corroborative evidence of tuberculosis from the sputum or X-rays.

This account of pulmonary radiography is incomplete by reason of the omission of all reference to appearances visible on the fluorescent screen. The omission is intentional, not because the subject is unimportant, but because the problems raised by such examinations cannot well be demonstrated apart from the presence of the patient, the dark room and the apparatus at work. While the value of the screen examination is fully recognised, it may be well to mention certain possible sources of error. It is usual to hear great stress laid on the significance of deficient movements of the diaphragm and poor illumination of the apices of the lungs as an early sign of pulmonary tuberculosis. It should be remembered that these two signs are in some degree present in practically every case of phthinoid chest, simply showing under what unfavourable conditions such a chest has to work. The signs, therefore, do not in themselves indicate the presence of pulmonary tuberculosis.

### DISCUSSION.

Dr. Bergin said that, while agreeing that peri-bronchial infiltrations are usually not indications of tuberculosis, he believed they were often indicative of early pathological changes. He did not agree that, if the upper level of fluid in a chest appeared as a line, gas was present ; it was exceedingly common to see the upper level in this way. He thought that one could sometimes definitely state after seeing a skiagram that a cavity was present, although none could be found clinically.

Dr. Mayes maintained that often X-ray diagnosis of tubercle could be made long before clinical signs enabled this to be done. He attached importance to diminution of diaphragmatic excursion; this meant a gross lesion of the lung. He emphasised the importance to be given to shadows in the outer third of the chest area; in the medial third shadows were not uncommon. The more dense and definite a shadow the less active the condition causing it.

Dr. Edgeworth described a case in which good health was interrupted by a sudden hæmoptysis. No physical signs appeared, except loss of diaphragmatic movement, revealed by skiagraphy. Three weeks later an apical friction was found. Sanatorium treatment was successful.

Dr. Symes thought that "skiagraphy shows too much, rather than too little." He had never seen a case with no symptoms or physical signs in which a skiagram helped a diagnosis of phthisis. He agreed that the diagnosis of cavities should not be made by skiagraphists, and thought they were inclined to see more on their films than existed in the patient. He was interested to learn of the tracheal deviation as a new and helpful sign, and asked whether there was any means of diagnosing adhesions by the X-ray.

Mr. Walters related a case in which a definite upper level of fluid was seen. No empyæma was found, but at operation a lung abscess was discovered.

Dr. Nixon regarded this method as of great importance in the *routine* examination of the chest, and sketched a systematic procedure for such examinations. Screening was important. He agreed that striæ did not necessarily indicate tubercle, and that " cavities " diagnosed by skiagraphy often did not exist at autopsy. But a similar mistake was not unknown when physical signs were relied on.

Dr. Carey Coombs thought that a linear upper border of fluid did appear when no physical signs indicated gas, and suggested that we might have to reconstruct our ideas on intra-thoracic physics ; this promised an interesting research. The great value of X-rays lay in their help in showing the position and extent of lesions.

Dr. Carleton, in reply, said he believed there was one sign of a cavity which was reliable, namely a semi-circular area of which the straight side was influenced by gravity, and which could be made circular by coughing up the contents. In regard to the line above fluid, he meant a clear-cut definite line, about which there was no mistake, this was pathognomonic of gas. When screening a chest the eyes should be fully accommodated to darkness, and minimal illumination was useful in bringing out slight differences in transparence. Defective movement of the diaphragm was a sign of pleurisy, not of tubercle. He wished to mention that artificial pneumothorax was the best method of arresting hæmoptysis.

## Figures and Tables

**Figure f1:**
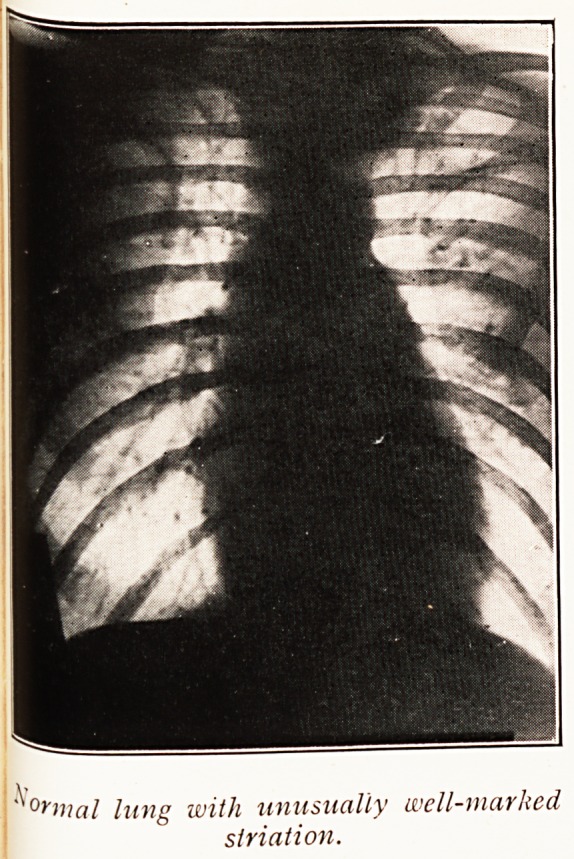


**Figure f2:**
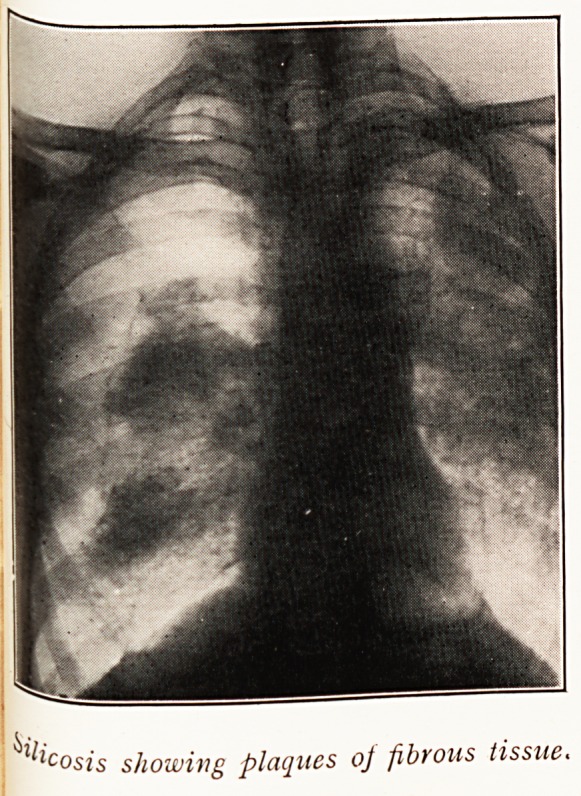


**Figure f3:**
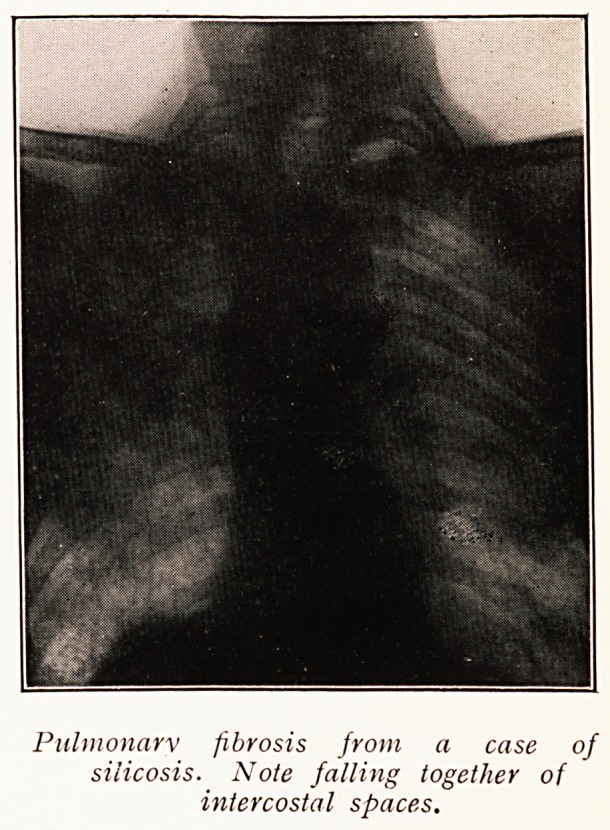


**Figure f4:**
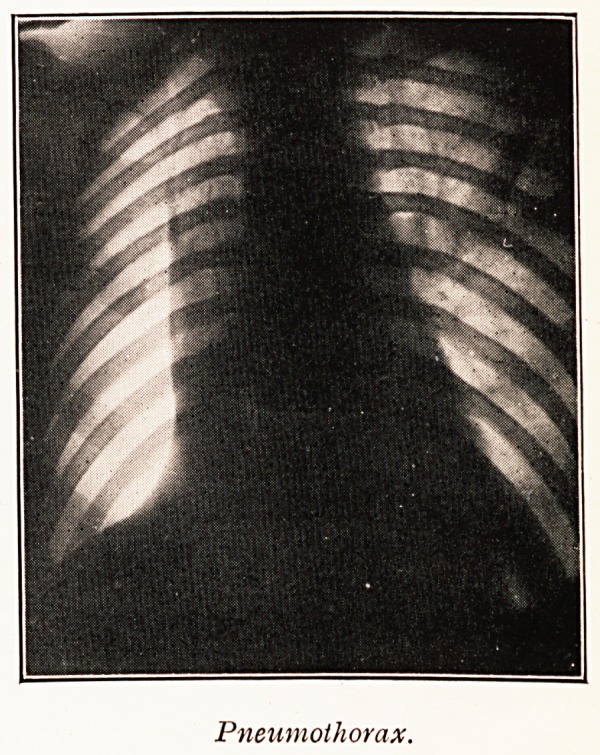


**Figure f5:**
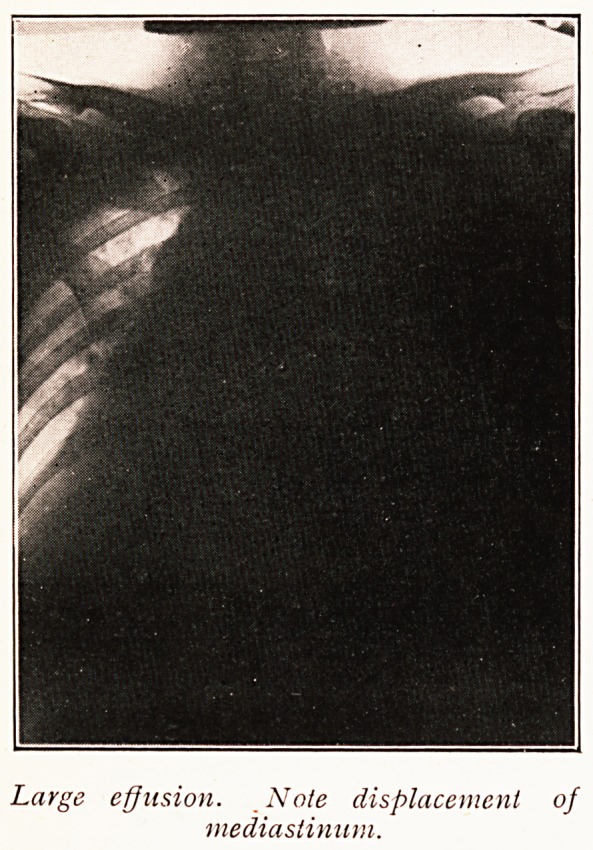


**Figure f6:**
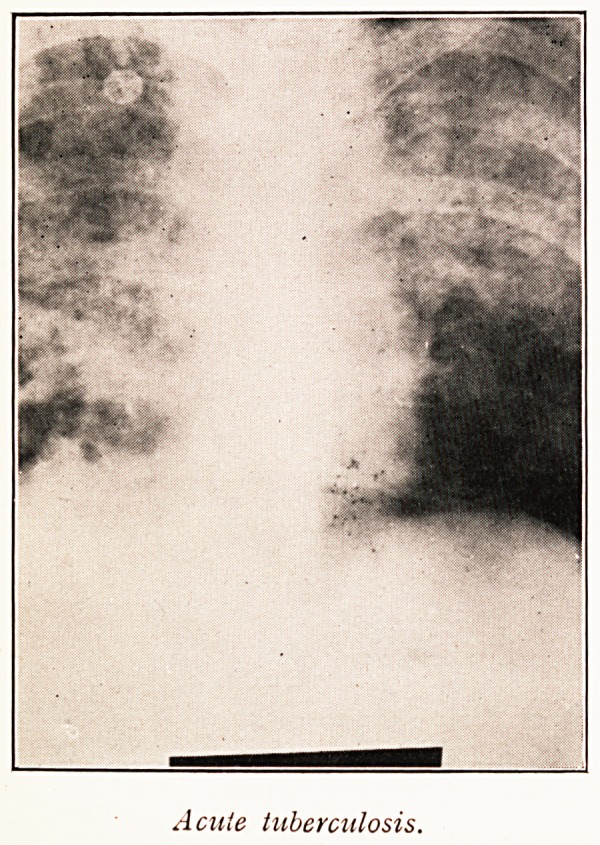


**Figure f7:**
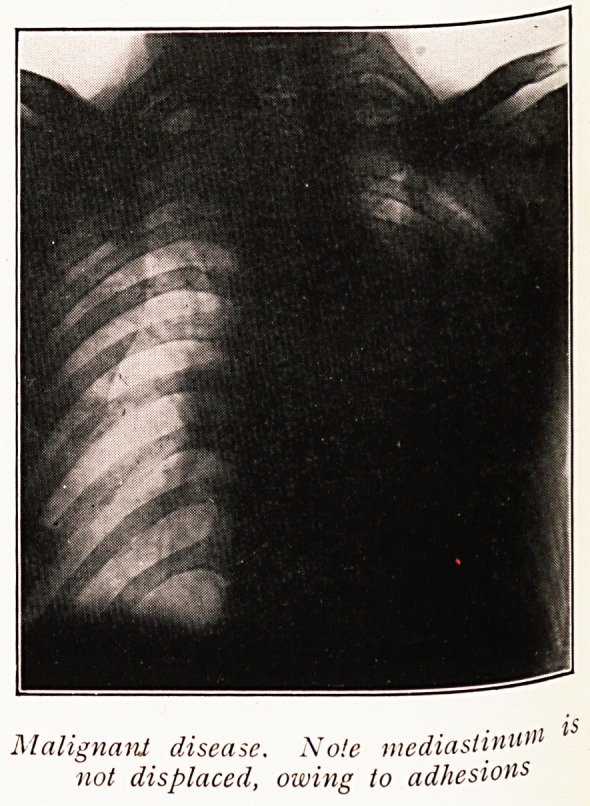


**Figure f8:**